# Chronic Care Model in Italy: a narrative review of the literature

**DOI:** 10.1017/S1463423621000268

**Published:** 2021-06-24

**Authors:** Fabio Petrelli, Giovanni Cangelosi, Giulio Nittari, Paola Pantanetti, Giulia Debernardi, Stefania Scuri, Getu Gamo Sagaro, Cuc Thi Thu Nguyen, Iolanda Grappasonni

**Affiliations:** 1 School of Medicinal and Health Products Sciences, University of Camerino, Camerino, Italy; 2 Asur Marche – Area Vasta 4 Fermo, Fermo, FM, Italy; 3 Ospedale Maggiore Ausl Bologna, Bologna, BO, Italy; 4 Department of Pharmaceutical Administration and Economics, Hanoi University of Pharmacy, Hanoi, Vietnam

**Keywords:** Chronic Care Model, COVID-19, health organization, narrative review, chronic diseases

## Abstract

**Aim:**

To analyze scientific literature on the development and implementation of the Chronic Care Model (CCM) in treating chronic diseases in the Italy context. Besides, to evaluate the effects of the activities carried out by the operators participating in the CCM on clinical care.

**Background:**

Italy is the second country globally for longevity, with 21.4% of citizens over 65 and 6.4% over 80. The CCM fits into this context, a care model aimed primarily at patients suffering from chronic diseases, especially in emergencies, as the recent COVID-19 pandemic.

**Methods:**

PubMed, Embase, Scopus, Cinahl, and Cochrane Library scientific databases were consulted, and the records selected as relevant by title and abstract by nine independent scholars, and disagreements were resolved through discussion. Finally, the studies included in this review were selected based on the eligibility criteria.

**Results:**

Twenty potentially relevant studies were selected, and after applying the eligibility criteria and screening by the Critical Appraisal Skills Program tool, eight included in this review. The studies showed the effectiveness of CCM for managing patients with heart failure in primary care settings and significant improvements in clinical outcomes, the reduction of inappropriate emergency room access for chronic patients, and the improvement of patients’ overall health with diabetes. The CCM organizational model is effective in improving the management of metabolic control and the main cardiovascular risk factors. Furthermore, this modality also allows doctors to dedicate more space to patients in the disease’s acute phase.

**Conclusion:**

The CCM, with its fundamental pillars of empowering self-management of care, could represent a valid alternative to health management. The managers of health services, especially territorial ones, could consider the CCM for the improvement of the treatments offered.

## Introduction

Globally in 2015, there were around 900 million older people, and that number will have doubled by 2050, and one in five people will be over 65 (World Health Organization, 2020b). Chronic diseases are strongly correlated with aging, and about 23% of the global disease burden occurs in people aged 60 and older (Prince *et al.*, 2015). For WHO, therefore, urgent action by national and international institutions is needed to achieve the global goals of reducing complications from the most common chronic non-communicable diseases such as heart, lung, neurology, cancer, and diabetes (World Health Organization, 2015). According to a 2014 international WHO report, non-communicable diseases such as cancer, diabetes, cardiovascular and respiratory diseases have caused the deaths of 38 million people worldwide, equal to 68% of the total deaths recorded (World Health Organization, 2015). Non-communicable diseases were responsible for 77% of the burdens of diseases and approximately 86% of premature death worldwide (World Health Organization, 2014). Promoting the improvement of protective factors and adopting healthy behaviors are important strategies for reducing the burden of non-communicable diseases (Nittari *et al.*, 2019; Ricci *et al.*, 2017, 2018). The governing and management bodies of assistance would have the ability to change this global trend, consequently reducing the people affected by these diseases and their comorbidities, by merely investing between 1 and 3 US dollars per person/year (World Health Organization, 2020b). Italy has the highest percentage of the elderly population in Europe (21.4% over 65 and 6.4% over 80) (Mazzola *et al.*, 2016). The elderly population are more likely to have chronic conditions (often multiple). In addition to having a natural mortality rate higher than the rest of the population, they may have increased chances of particularly disabling complications that would indirectly affect the entire community to which they belong. For this reason, combating chronic diseases is a public health priority in both wealthier and low-income countries (World Health Organization, 2015).

Investing in chronic disease prevention and control, reducing risk at both the individual and community level, and focusing the intervention on those at high-risk people will improve quality of life and save money related to healthcare (World Health Organization, 2017). Today, most health systems are designed to address acute conditions, which require mainly rapid and particular interventions, but may be unsuitable for managing chronic diseases and their complications (World Health Organization, 2017). To reduce the burden of chronic disease on healthcare, assistance models that mobilize (or redirect) significant economic and professional resources directly to the region would be needed. In addition to implementing primary prevention programs, it could limit the damage from relapse, worsening, and disability by initiating early treatment, preventing disease progression, and promoting proper healing and appropriate handling through the Chronic Care Model (CCM) framework. Therefore, the institutions’ objective should be to reduce the impact of chronic diseases, bringing quality and life expectancy to acceptable levels in Italy and all other European countries. According to the new WHO guidelines report, integrated care for older people through community-based services effectively prevents, slow, or reverses the decline in the most vulnerable population with limited physical and mental abilities (World Health Organization, 2017). The CCM fits into this context, a care model explicitly aimed at patients suffering from particularly disabling diseases such as diabetes, heart failure, lung failure, or cancer (Bodenheimer, Wagner, and Grumbach, 2002; Petrelli *et al.*, 2020). Developed by Professor Wagner and his colleagues at the McColl Institute for Healthcare Innovation in California, it suggests a “proactive” approach based on the principle that patients become an integral part of their care process (Bodenheimer *et al.*, 2002). This articulated system of care presupposes a change in the classic healthcare paradigm that passes from a mainly passive welfare to an active self-care, or from the Traditional Sickness Care Model to the CCM (Bodenheimer *et al.*, 2002; Martin and Peterson, 2008; Wagner, 2000; Coleman *et al.*, 2009). The CCM is an organizational approach to managing people with chronic illnesses in a primary care setting. The system is population-based and creates practical, supportive, and evidence-based interactions between an informed and active patient and a trained, proactive practice team. While the traditional sickness care model is disease-based using a biomedical approach to problems, symptoms elicited by patients are complied with. The CCM, therefore, proposes a series of changes at all levels of healthcare systems assistance, capable of directly improving the conditions of the chronically ill and indirectly the management of economic and community resources (Bodenheimer, Wagner, and Grumbach, 2002). In detail, the CCM is characterized by six fundamental components (Table 1) (Bodenheimer *et al.*, 2002; Martin and Peterson, 2008; Wagner, 2000; Coleman *et al.*, 2009).

**Table 1.** Fundamental components of Chronic Care Model

Resources community	Quality of care	Support for self-care	Organization in specific teams	Evidence-based guidelines	Efficient and modern information structures
Direct interaction with stakeholders (volunteer groups, self-help groups, centers for the elderly, third sector in general)	Innovative introduction into care processes	Direct patient focus on all self-care and educational interventions	Mainly general practitioner, specialist doctors from specifically trained nurses	Support for evidence-based clinical and care decisions and evidence-based practice	Integration and sharing of care information between all the actors involved in the articulated care process

According to the CCM, informing patients and providing them with proper self-care support is a process of fundamental importance for achieving the best possible state of health, which, specifically in subjects suffering from chronic diseases, can be maintained even in the absence of continuous medical assistance (Epping-Jordan, 2004). Recently, prestigious international scientific communities such as the American Diabetes Association and the American College of Cardiology have suggested the CCM for the management and assistance of two of the most common chronic diseases and the main related risk factors (Carey *et al.*, 2018; American Diabetes Association, 2019). The CCM is designed to improve patients’ health status, especially patients with chronic illnesses, by transforming everyday care into proactive, planned, and population-based care. It is a framework in which healthcare providers translate the general idea of change into specific, frequent and locally distinctive applications. Therefore, the specific practice changes correlated with the elements of CCM vary from organization to organization as well as from country to country. Accordingly, the specific means of implementing CCM components influence the likelihood of improvement in outcomes. Besides, CCM implementation is beneficial in patient adherence with therapy, promotion of healthy behaviors, satisfaction with clinical care, and reduced medical burden (Yeoh *et al.*, 2018). After Japan, Italy is the second-highest country globally by the proportion of the elderly population (22.4% of the total population was estimated to be aged 65 years and older in 2015) (IIASA, 2018), and older adults are more prone to chronic diseases. The present study aimed to evaluate the development and implementation of the CCM in Italy, specifically the clinical care and quality outcomes of the pathologies with the highest impact of chronicity, such as diabetes, heart disease, and primary care general, were analyzed.

## Materials and methods

The review was conducted with the preliminary development of a search protocol through specific PICOS and search strings updated as of 31 December 2019 on scientific databases, including PubMed, EMBASE, Scopus, CINAHL, and Cochrane Library. Consequently, 20 relevant studies were identified, exclusively in full English or Italian text. The full text was independently assessed by nine investigators (FB, GC, GN, PP, GD, SS, GGS, CTTN, and IG), and disagreements were resolved through discussion. The selected studies were critically appraised using CASP (Critical Appraisal Skills Program) (CASP, 2018) and Equator guidelines (Mariona *et al.*, 2015). Finally, eight primary studies were selected according to the eligibility criteria (Table 2).

**Table 2.** PICOS research

**P**: chronic diseases such as diabetes, cardiac, pulmonary (COPD, dyspnea, or asthma), hypertension, chronic renal diseases or renal insufficiency, neurological (central, ischemic, or hemorrhagic), and oncological in Italy
**I**: interventions according to the CCM organizational conditions
**C**: traditional treatments or different CCMs versus cures through the assumptions of
**O**: clinical care outcomes
**S**: primary research study

### Inclusion and exclusion criteria

Studies intended to assess or manage patients under CCM conditions were included. In other words, studies mentioning the development and application of CCM through community resources (the third sector in general), care processes, self-care, describing the organization’s contribution to the specific team (general practitioner, specialists, and/or specifically trained nurses), reference to evidence-based guidelines, an efficient and modern information structure, studies published in Italian or in English, peer-reviewed journals or articles, studies published as abstract or thesis from the database as mentioned above describing CCM application, and studies published up to 31 December 2019 have been included in this review.

## Results

Of the 20 studies, eight articles remained ultimately based on the eligibility criteria, and the summary result is presented in Table 3.

**Table 3.** Summary of the results and characteristics of the included studies

Study ID	Region	Setting	Study design/population	Main interventions	Main outcomes	Study rank
Buja *et al.* (2019)	Lombardia/Toscana/Sicilia	Primary medicine/diabetic patients	Almost experimental/GP	Observation of five quality indicators for adherence to new models of care for chronic patients	The total score of quality indicators before and after the CCM experimentation of the ASL Arezzo GPs: creatinine+5.59, microalbuminuria +14.44, HbA1c + 9.18, lipid profile +5.33, treatment with statins +3.20	Medium
Ballo *et al.* (2018)	Toscana	Primary care patients with heart failure	Retrospective cohort study	Creation of specific clinical assistance paths, therapeutic counseling and/or for lifestyle improvement	713 hospitalizations in 432 patients in the CCM group and 1135 in 657 users in the control group: 632 deaths in the CCM group and 1393 in the control group	Medium
Robusto *et al.* (2018)	Puglia	Patients with chronic non-oncological conditions	Retrospective cohort study	Support for counseling, follow-up organization, telemedicine and specific IT resources	CCM is associated with less recourse to unplanned hospitalizations than in the control group (IRR 0.92; 95% CI 0.91–0.92)	Medium
Musacchio *et al.* (2018)	Lombardia	Diabetic patients	Multicenter randomized controlled trial	Application of the Sinergia 19 model focused on self-care and support to the specialist care of specific CCM teams	Primary end point, after 12 months, HbA1c levels were reduced by 0.47% in the experimental group and by 0.32% in the control group	High
Barletta *et al.* (2016)	Toscana	Primary care	Controlled study	Multidisciplinary teams, self-care education, specific clinical pathways, IT support, and counseling for healthy lifestyles	After 1 year, the indicator of adherence to the Secondary Prevention Guidelines (GDI) 22 grew by 8.1%; during the second year, it showed a further increase of 1.6%	Low
Seghieri *et al.* (2016)	Toscana	Diabetic patients	A retrospective cohort study	Observation of the occasional diagnosis of diabetes in hospitalized patients in the Tuscany Region in 2011	974 new cases of unrecognized diabetes: 834 traditional primary care and 140 experimental CCM: standardized IT per 100 000 inhabitants or hospitalized patients 383.3 (357.2–409.4) no CCM and 289.4 (237.9–340.9) CCM, RR 0.75 (0.63–0.91) CCM	Low
Profili *et al.* (2017)	Toscana	Diabetic patients resident in Tuscany	Retrospective cohort study	A multidisciplinary team that managed the patient in education and support for care according to an individualized therapeutic plan and establishing regular follow-up of primary and secondary prevention	CCM protective effect for long-term neurological complications (RTI 0.85; 95% CI 0.76–0.95), acute long-term cardio-cerebrovascular (RTI 0.81; 95% CI 0.71–0, 92) and mortality (HR 0.88; 95% CI 0.81–0.96)	Medium
Musacchio *et al.* (2011)	Lombardia	Patients with non-decompensated diabetes in Cusano-Milanino territorial care	Randomized controlled trial	After the first diabetic visit and meeting with nurses and dieticians, the individualized therapeutic plan was drawn up. The Synergy program includes self-care, use, and support of telemedicine and follow-ups managed by nurses and dietitians with a view to continuous improvement	A total of 1004 patients were included. After 12-month follow-up, the percentage of patients with HbA1c ≤ 7.0% (≤53 mmol/mol) increased from 32.7% to 45.8% (*P* < 0.0001), while those with HbA1c ≥ 9% (≥75 mmol/mol) decreased from 10.5% to 4.3% (*P* < 0.0001). Patients with LDL < 100 mg/dL (<2.59mmol/l) increased from 40% to 47% (*P* < 0.0001), while those with LDL ≥ 130 mg/dL (≥3.36 mmol/l) decreased from 26.6% to 19.7%	Medium

### Heart failure

Ballo *et al.* (2018) evaluated the impact of CCM treatment on patients with heart failure in the primary care setting. The same study found that the nurse played a key role in the project responsible for the crucial stages of the care process. The exposed cohort was represented by all the patients with a certain diagnosis of heart failure who were assisted by GPs adhering to the CCM project. In contrast, the unexposed was described by patients with the same diagnosis but followed by non-adherent GPs: 1761 cases and 3522 controls. During the observation, 713 hospitalizations for heart failure occurred in 432 patients in the CCM group (12.1 events per 100 patient-years) and 1135 hospitalizations in 657 subjects in the control group (10.3 events per 100 patient-years) (Ballo *et al.*, 2018). This indicates a higher incidence rate in the CCM group compared to controls (Ballo *et al.*, 2018) of patients with heart failure. Similarly, the CCM group had a longer mean length of hospitalization than the control groups (8.8 days versus 8.1 days) (Ballo *et al.*, 2018). The same study revealed that CCM was independently associated with a 35% higher hospital admission chance (Ballo *et al.*, 2018).

The study compared the rates of planned and urgent hospitalizations, and a significantly higher rate of planned hospitalizations than urgent hospitalizations was noticed (Ballo *et al.*, 2018). In the 4-year follow-up, fewer deaths were recorded in the CCM group than in the control group (632 deaths versus 1393 deaths) (Ballo *et al.*, 2018). After specific hospitalization for heart failure, patients treated with CCM still had a 16% lower risk of death than controls (Ballo *et al.*, 2018). In this regard, the management of patients with heart failure by CCM has led to a decrease in mortality and an increase in hospitalizations, possibly attributable to the effectiveness of the primary care provided, which may promote patients’ overall survival with heart failure.

### Patients with chronic non-oncological conditions

The retrospective cohort study was conducted in six local health units; those participated in the development and implementation of Puglia Care (Robusto *et al.*, 2018). This study reported that a case manager played a crucial role in the Puglia Care program by coordinating the communication and information process among the stakeholders involved. One thousand seventy-four cases and 2126 controls cohorts represented the study. In the Puglia Care program’s intervention group during the pre-inclusion and follow-up periods, admissions are almost similar rather than cost differences. Regarding the costs of direct care, there has been a significant reduction in the costs of unplanned hospitalizations. However, the total costs incurred for hospitalizations, medications, and specialist ambulatory visits were significantly increased (Robusto *et al.*, 2018). In contrast, for the control group during the follow-up period, hospitalization is almost twice as high as unplanned hospitalization during the pre-inclusion period (Robusto *et al.*, 2018). Regarding incidence, unplanned hospitalizations during the pre-inclusion period of the intervention group had a higher incidence rate than the usual care group period.

Furthermore, by applying the CCM for non-acute cases, reducing the number of unscheduled hospitalizations is possible. The study conducted in the Puglia Care program reported that the number of unplanned hospitalizations significantly decreased (Robusto *et al.*, 2018). The lower recourse to the Puglia Care group’s emergency services during the follow-up was also evidenced by the reduction in healthcare expenses in unplanned hospitalizations (Robusto *et al.*, 2018). However, drug costs and expenses for specialist outpatient visits had increased, although using CCM to treat patients with chronic diseases (Robusto *et al.*, 2018). Hence, the implementation of the CCM could favor the reduction of improper access to the emergency room by chronic patients and that the increases in drugs and visits to the most fragile subjects can be attributed to the organization of more efficient routes.

### Professionals involvement in CCM care

The study carried out in the Tuscany region of Italy had considered GPs involved in the treatment process through the CCM by comparing before and after joining the CCM (Barletta *et al.*, 2016). Hence, the study has been reported that there was greater adherence to the Secondary Prevention Guidelines in the two experimental groups. The group joined in 2010 went from 31.3% of the pre-observation period to 42.4% 3 years later. Also, the 2011 group went from 33.8% to 41.5%, while the control group went from about 29% to just over 32% at the end of the observation (Barletta *et al.*, 2016). There was a significant increase in both intervention groups, especially after the first year of experimentation: from 31.3% in 2009 to 41.7% in 2011 for the group that joined in 2010 and from 34.9% in 2010 to 41.5% in 2012 for GPs who joined the following year (Barletta *et al.*, 2016). No differences were observed between the three GP groups for adherence to statin therapy after 3 years: from 27.8% pre-inclusion to 35.0% for the 2010 group, from 27.8% to 33.3% for the 2011 group, and from 26.5% to 33.4% for the control group (Barletta *et al.*, 2016). Thus, GPs adhering to the CCM performed better than the control group of non-CCM members.

The study was conducted in the northern part (Lombardy, Veneto, and Emilia Romagna), two central (Tuscany and Marche), and like many southern (Sicily and Puglia) of Italy by considering the GPs of a single local health authority (ASL) for each region (Buja *et al.*, 2019). The same study considered proactive models for the organization of primary care adopted heterogeneously at the national level (Buja *et al.*, 2019). Accordingly, CCM for the Tuscany region (Arezzo), the Chronic Related Groups Model for Lombardy (Bergamo), and Integrated Ambulatory Care Model for the Sicily region (Ragusa) (Buja *et al.*, 2019). The study evaluated GPs’ adherence to the new organizational model tested through specific pre- and post-intervention quality scores. Consequently, the CCM showed evident increases in the total adherence score of the GPs of the indicators considered, including creatinine, microalbuminuria, HbA1c, lipid profile, and treatment with statins. The study demonstrated that introducing new proactive primary care models could ensure the sound management of chronic diseases in primary care (Buja *et al.*, 2019).

### Diabetes

A control clinical trial study was conducted to compare with CCM and usual care concerning education in the self-management of blood glucose (Musacchio *et al.*, 2018). The duration of the experimental study with CCM was 12 months. After 12 months of the follow-up, HbA1c levels in the CCM group showed a significant reduction compared to the usual care group (0.47% versus −0.32%) (Musacchio *et al.*, 2018). Furthermore, after 24 months, the average reduction of HbA1c levels in the CCM group was two times higher than that of the usual care group (0.39% versus 18%) (Musacchio *et al.*, 2018). In general, the self-care program had more significant effects on HbA1c over time than in a short period. In this respect, the CCM could be more effective by self-management of blood glucose levels.

The study by Seghieri *et al.* (2016) observed all diagnoses of occasional diabetes in patients admitted to hospitals in the Tuscany Region in 2011 treated by GPs with CCM or with traditional treatments. Out of 214 991 hospital discharges, 974 new cases of previously unrecognized diabetes were diagnosed: 834 in traditional primary care medicine and 140 in that adhering to the CCM territorial trial with a standardized IT per 100 000 inhabitants or hospitalized patients equal to 383.3 (357.2–409.4) for conventional primary care and 289.4 (237.9–340.9; RR 0.75, 0.63–0.91) for CCM-treated patients (Seghieri *et al.*, 2016). Even GPs’ adherence to the CCM did not show significant differences depending on the territorial area of reference (Seghieri *et al.*, 2016). A greater risk of an accidental diabetes diagnosis was observed in non-Italian origin (Seghieri *et al.*, 2016).

Profili *et al.* (2017) conducted a study on patients with type two diabetes mellitus in the Tuscany region of Italy. The doctors who joined the CCM-based program treated 14 016 diabetic patients, of which 8574 (61.2%) were enrolled in the CCM group and 5442 were treated with traditional care. Significant improvements were observed in the CCM group for adherence to the Complication Prevention Guidelines with a ratio of incidence rates (IRR: 1.58) and for cardiovascular complications in the long term (IRR: 1.11) (Profili *et al.*, 2017). A protective effect was also observed for patients managed with CCM for long-term neurological complications and cardio-cerebrovascular complications. Despite a direct increase in costs, CCM can improve the diabetic patient’s general health and promote more effective management of possible medium and long-term complications (Profili *et al.*, 2017).

Musacchio *et al.* (2011) reported that the CCM organizational model was effective in improving the management of metabolic control and significant cardiovascular risk factors. For example, chronic patients after 12-month follow-up ranging from 6 to 24 months, the percentage of subjects with HbA1c ≤ 7.0% (≤53 mmol/mol) increased from 32.7% to 45.8% while that with HbA1c ≥ 9% (≥75 mmol/mol) decreased from 10.5% to 4.3% (20). Also, users with LDL cholesterol <100 mg/dl (<2.59 mmol/l) increased from 40% to 47% while those with LDL cholesterol ≥ 130 mg/dl (≥3.36 mmol/l) decreased from 26.6% to 19.7% (Musacchio *et al.*, 2011). Thus, the CCM allows clinicians to devote more space to patients in the disease’s acute phase.

## Discussion

In this review, heart failure, diabetes, and non-oncological conditions, clinical care, and outcomes through the CCM were evaluated. Also, professional involvement in the treatment process via CCM was also assessed. Accordingly, among the eight studies included in this review, one study showed the effectiveness of CCM in managing patients with heart failure in primary care settings and significant improvements in clinical outcomes (Ballo *et al.*, 2018). Another study reported that using CCM for chronic non-oncologic cases could reduce inappropriate emergency room access for chronic patients as well as significantly reduce the number of unscheduled hospitalizations (Robusto *et al.*, 2018). In the other four studies, an improvement in clinical outcomes indicated that CCM could refine patients’ overall health with diabetes and long-term complications (Musacchio *et al.*, 2011; 2018; Seghieri *et al.*, 2016; Profili *et al.*, 2017). Furthermore, studies reported that professional involvement in CCM implementation contributes to improving clinical care and ensuring good management of chronic conditions in primary care (Buja *et al.*, 2019; Barletta *et al.*, 2016). However, the included studies’ authors mentioned that various limitations were present when studying CCM implementation, clinical care and outcomes, and patients’ enrollment in the CCM program. Among eight studies included, one study reported the short follow-up to measure the outcomes, absence of adherence to therapy, and discharge plan (Ballo *et al.*, 2018). The other study reported no quality indicators established at the center to evaluate the program (Robusto *et al.*, 2018); one study reported selection bias in patient enrollment, significant differences in outcomes, and different attitude in treatment adherence of patients (Profili *et al.*, 2017). A study reported limited randomization, especially lack of control group to evaluate the effectiveness of the model (Musacchio *et al.*, 2011).

The aging of the Italian population and the consequent need to respond to increasingly articulated and complex health needs is one of the objectives that every health organization is committed to pursuing from a modern perspective of effectiveness and efficiency. The Italian “National Chronicity Plan” (Ministry of Health, 2016) identifies the CCM as the reference organizational model for managing chronic diseases. The review emerged that in Italy, only the Tuscany Region has fully adopted this system for the health management of chronic diseases since the 2008–2010 Regional Plan (Barletta *et al.*, 2008). In this context, the retrospective analysis of the data as well as the randomized clinical trials could evaluate the CCM as applicable to the Italian National Health System, especially at the level of primary care. An elderly population such as the Italian one, which will age more and more in the next two decades, will hopefully have to be assisted through the management and organizational tools such as to be able to maintain high levels of quality of life.

The CCM, with its fundamental pillars of empowering self-management of care, could represent a valid alternative to health management. The managers of health services, especially territorial ones, could therefore consider the CCM for the process of improving the care offered. Also, the recent COVID-19 pandemic – declared by the World Health Organization in March 2020 (World Health Organization, 2020a) – has emphasized the continuation of the treatment of all chronic diseases even in delicate phases such as a pandemic of this magnitude. Italy, with 239 627 cases, 33 498 deaths, and 29 282 health workers who tested positive (data as of 22 June 2020), is one of the most affected countries in the world (Higher Institute of Health (ISS2020)). The inevitable overhaul of chronic disease organizational processes will undoubtedly be one of the major challenges the country will face in short to medium term. It will not compromise public health and the economic support necessary to satisfy an increasingly pluralistic care population. Investments that place the patient at the center of the entire care process, such as CCM, could be decisive for improving the overall health picture of the entire Italian population, especially after the necessary reorganization of the post-COVID-19 regional care system.

There are some limitations to this review. This review was mainly conducted in Italy. The conclusion may not be generalized to other European Union countries due to various circumstances such as age variation and other socio-demographic characteristics. The review also considered limited chronic conditions, and other reviews should consider cardiovascular disease and other chronic diseases’ clinical care and outcomes via CCM.

### Conclusion

The CCM is an effective way to manage patients with chronic conditions, especially patients with heart failure, diabetes, and other non-oncological conditions. According to the present narrative review, the CCM implementation in the primary care setting can substantially improve the clinical outcomes, enhance patients’ life quality, and reduce unscheduled hospitalization. Healthcare professional involvement in producing and leading the model’s implementation is paramount significant for effective CCM implementation and quality clinical care through CCM. Therefore, professional participation, patient enrollment, follow-up of recruited patients, evaluation of CCM efficacy in primary care using the quality indicator, on-site training for work team members, and applications of CCM components need to be carefully addressed to ensure sustainable CCM implementation and improve quality of clinical care in primary care facilities.
